# Naringenin Inhibition of the *Pseudomonas aeruginosa* Quorum Sensing Response Is Based on Its Time-Dependent Competition With *N*-(3-Oxo-dodecanoyl)-L-homoserine Lactone for LasR Binding

**DOI:** 10.3389/fmolb.2020.00025

**Published:** 2020-02-28

**Authors:** Sara Hernando-Amado, Manuel Alcalde-Rico, Teresa Gil-Gil, José R. Valverde, José L. Martínez

**Affiliations:** ^1^Departamento de Biotecnología Microbiana, Centro Nacional de Biotecnología, Consejo Superior de Investigaciones Científicas, Madrid, Spain; ^2^Laboratorio de Genética e Inmunología Molecular, Instituto de Biología, Facultad de Ciencias, Pontificia Universidad Católica de Valparaíso, Valparaíso, Chile; ^3^Millennium Nucleus for Collaborative Research on Bacterial Resistance, Valparaíso, Chile; ^4^Servicio de Computación Científica, Centro Nacional de Biotecnología, Consejo Superior de Investigaciones Científicas, Madrid, Spain

**Keywords:** quorum sensing, virulence inhibitor, LasR, naringenin, *Pseudomonas aeruginosa*

## Abstract

Bacterial quorum sensing (QS) is a cell-to-cell communication system that governs the expression of a large set of genes involved in bacterial–host interactions, including the production of virulence factors. Conversely, the hosts can produce anti-QS compounds to impair virulence of bacterial pathogens. One of these inhibitors is the plant flavonoid naringenin, which impairs the production of QS-regulated *Pseudomonas aeruginosa* virulence factors. In the present work, we analyze the molecular basis for such inhibition. Our data indicate that naringenin produces its effect by directly binding the QS regulator LasR, hence competing with its physiological activator, *N*-(3-oxo-dodecanoyl)-L-homoserine lactone (3OC12-HSL). The *in vitro* analysis of LasR binding to its cognate target DNA showed that the capacity of naringenin to outcompete 3OC12-HSL, when the latter is previously bound to LasR, is low. By using an *E. coli* LasR-based biosensor strain, which does not produce 3OC12-HSL, we determined that the inhibition of LasR is more efficient when naringenin binds to nascent LasR than when this regulator is already activated through 3OC12-HSL binding. According to these findings, at early exponential growth phase, when the amount of 3OC12-HSL is low, naringenin should proficiently inhibit the *P. aeruginosa* QS response, whereas at later stages of growth, once 3OC12-HSL concentration reaches a threshold enough for binding LasR, naringenin would not efficiently inhibit the QS response. To test this hypothesis, we analyze the potential effect of naringenin over the QS response by adding naringenin to *P. aeruginosa* cultures at either time zero (early inhibition) or at stationary growth phase (late inhibition). In early inhibitory conditions, naringenin inhibited the expression of QS-regulated genes, as well as the production of the QS-regulated virulence factors, pyocyanin and elastase. Nevertheless, in late inhibitory conditions, the *P. aeruginosa* QS response was not inhibited by naringenin. Therefore, this time-dependent inhibition may compromise the efficiency of this flavonoid, which will be effective just when used against bacterial populations presenting low cellular densities, and highlight the importance of searching for QS inhibitors whose mechanism of action does not depend on the QS status of the population.

## Introduction

Quorum sensing (QS) is a cell-to-cell communication system that enables to coordinate a global response of a bacterial population when a certain cell density is reached (Nealson et al., 1970; Fuqua et al., 1994). This response is based on the production of intercellular communication molecules, which function as autoinducer signals (AIs) and are constantly synthesized and released by the bacterial population. The whole population perceives these AIs through their binding to their cognate receptors, which are then activated to globally regulate the QS response. The progressive accumulation of the regulator-AIs complexes promotes the AIs synthesis, thus providing a positive feedback to the system, and the subsequent modification of the expression of a large set of QS-regulated genes (Williams et al., 2007). Consequently, while at low cell densities the concentration of the AIs is very low, at higher cell densities, the signal concentration exceeds a given threshold and the QS response is exponentially activated.

The molecular mechanisms underlying the QS response have been deeply studied in *Pseudomonas aeruginosa*. In this opportunistic pathogen, the QS regulatory network is based on the synthesis and perception of the AIs *N*-acyl-homoserine lactones (AHLs) and 2-alkyl-4-quinolones (AQs) (Williams et al., 2000, 2007). In particular, *N*-(3-oxo-dodecanoyl)-L-homoserine lactone (3OC12-HSL) and *N*-butanoyl-L-homoserine lactone (C4-HSL) AHL signals are synthesized by the AHL synthases LasI and RhlI, respectively. The *lasR* and *rhlR* genes, which encode the receptors of 3OC12-HSL and C4-HSL respectively, are located upstream the *lasI* and *rhlI* genes. These two LuxR-type transcriptional regulators contain both, a DNA binding domain and a ligand binding domain (Lamb et al., 2003; Bottomley et al., 2007) and control the expression of hundreds of genes in *P. aeruginosa* (Wagner et al., 2003; Schuster and Greenberg, 2007). It is worth noting that several biological processes with relevance for *P. aeruginosa* infection, such as motility, production of virulence factors and biofilm formation, are regulated by the QS response (Parsek and Greenberg, 1999; Erickson et al., 2002; Bjarnsholt and Givskov, 2008). Indeed, it has been described that the disruption of the QS response reduces *P. aeruginosa* virulence (Tan et al., 1999; Diggle et al., 2007) and impairs biofilm formation (Wagner and Iglewski, 2008). This indicates that QS is a fundamental element in the success of this opportunistic pathogen for colonizing and infecting its hosts, from plants to humans. For those reasons, the QS system transcriptional regulators, such as LasR, have become attractive targets to be inhibited (Williams et al., 2000; Hurley et al., 2012; Kalia et al., 2017).

Plants have evolved different strategies to deal with bacterial infections, being inhibition of the QS response one of their potential defense mechanisms (Diggle et al., 2007). Therefore, plants have been widely screened in the search of compounds able to interfere with the QS response of bacterial pathogens (Rudrappa and Bais, 2008; Smyth et al., 2010; Vandeputte et al., 2011; Rasamiravaka et al., 2013; Corral-Lugo et al., 2016; Fernandez et al., 2018), particularly for those, as *P. aeruginosa*, able of infecting both humans and plants (Rahme et al., 1995). Among those potential inhibitors, naringenin seems to be particularly interesting (Salehi et al., 2019). This plant-produced flavonoid inhibits the inflammatory response (Jin et al., 2017). In addition, studies using animal models have shown that naringenin might have therapeutic potential for the treatment of different inflammation-related diseases, including sepsis and endotoxic shock, hepatitis, pulmonary fibrosis, radiation-induced lung injury, atherosclerosis, obesity, diabetes, or cancer (Zeng et al., 2018). Besides these effects in the host response, it has also been described that naringenin reduces the production of AHLs in *P. aeruginosa*, as well as the expression of QS-regulated genes, thus inhibiting the production of QS-regulated virulence factors, such as pyocyanin or elastase (Vandeputte et al., 2011; Rasamiravaka et al., 2013). Nevertheless, the mechanisms underlying naringenin-mediated inhibition of the QS response are still unknown. Herein, we analyze the interaction of naringenin with LasR, a master regulator of QS in *P. aeruginosa* and find out that this flavonoid competes with 3OC12-HSL for the binding of the nascent LasR protein. However, the inhibitory capacity of naringenin is limited when 3OC12-HSL has already bound the regulator. These results shed light on the mechanisms of QS inhibition based on targeting the LasR regulator in *P. aeruginosa* populations and may help in the design and development of efficient QS-inhibitors.

## Materials and Methods

### Bacterial Strains, Plasmids, and Growth Conditions

Bacterial strains and plasmids used during this work are listed in Table 1. Bacteria were routinely grown at 37°C in Lysogeny Broth, Lennox (LB) (Pronadisa) at 250 rpm. For the culture of the LasR-based reporter strain (Table 1), tetracycline 5 μg/mL was added to LB medium, while ampicillin 100 μg/mL was added in the case of the SHA011 LasR producing *E. coli* strain. When necessary, naringenin and/or 3OC12-HSL were added to *E. coli* and *P. aeruginosa* cultures. Both compounds were purchased from Sigma Aldrich and dissolved in dimethyl sulfoxide (DMSO).

**TABLE 1:** Bacterial strains and plasmids used in this work.

Bacterial strains		Description	Reference/origin
*E. coli* strains	BL21(DE3)pLysS	*hsdS gal (lcIts857 ind1 S am7 nin5 lacUV5-T7 gene 1)*	Sambrook and Russell, 2001
	SHA010	BL21(DE3)pLysS strain carrying pGEX6p	This work
	SHA011	BL21(DE3)pLysS strain carrying pSHA-LasR	This work
	JM109 (pSB1142)	LasR-based reporter strain	Miguel Cámara laboratory
*P. aeruginosa* strains	PAO1	Wild-type *P. aeruginosa* strain	Laboratory collection

**Plasmids**		**Description**	**Reference/origin**

pGEMT-Easy Vector		Amp^R^, commercial vector	Promega
pGEMT-*lasR*		pGEMT-Easy Vector carrying *lasR* gene from *P. aeruginosa* PAO1 strain.	This work
pGEX6p		Expression vector, Amp^R^	GE Healthcare
pSHA-LasR		pGEX6p vector carrying *lasR* gene from *P. aeruginosa* PAO1 strain.	This work
pSB1142		Reporter plasmid carrying the *lasR* gene and the *luxCDABE* operon under the control of the *lasI* promoter.	Miguel Cámara Laboratory

### Overexpression and Purification of LasR

The *lasR* open reading frame was amplified with the Expand Long Template System (Roche). PCR was carried out using the primers lasR-pGEX.fw and lasR-pGEX.rv (Table 2) with the following conditions: 2 min at 94°C, 35 cycles of 15 s at 94°C, 30 s at 55°C, 1 min at 68°C and 7 min of final elongation at 68°C. The DNA fragments amplified were purified following the supplier’s instructions with the QIAquick Purification PCR Kit (Qiagen) and cloned into pGEMT-Easy vector. The cloned *lasR* was sequenced to verify that no mutations were introduced during PCR process. This plasmid (pGEMT-*lasR*), containing the gene amplified from lasR-pGEX.fw/lasR-pGEX.rv, was digested with *Eco*RI-*Bam*HI and the DNA product was cloned into the polylinker site of pGEX6p, immediately after the GST gene (pSHA-LasR). The resulting plasmid was introduced by transformation in *E. coli* BL21. The resulting clone, which encodes a GST–LasR fusion protein, was dubbed *E. coli* SHA011, and the respective one containing pGEX6p without LasR, SHA010 (Table 1). For the induction of LasR production, cells were grown with 1 mM IPTG at 17°C overnight. LasR was purified as a GST-fusion protein according to the manufacturer’s instructions for the GST gene fusion system (Amersham Pharmacia Biotech, Europe, GmbH) using an immobilized Glutathione Sepharose column. After elution, the GST affinity tag was removed in solution with PreScission protease (GE Healthcare). Separation of LasR and the GST was done using a Glutathione Sepharose column. After dialysis against TBS (20 mM Tris-HCl, 200 mM NaCl, pH8), the purified native protein appeared as a single band on SDS-PAGE. The verification that the purified protein was LasR was performed by matrix-assisted laser desorption ionization-time of flight (MALDI-TOF) mass spectrometry at the Proteomics Service of the Centro Nacional de Biotecnología.

**TABLE 2:** Primers used in this study.

Primer	Sequence	Utilization
rplU.fw	5′-CGCAGTGATTGTTACCGGTG-3′	Real time PCR for *rplU* and for checking DNA in RNA samples
rplU.rv	5′-AGGCCTGAATGCCGGTGATC-3′	
lasA.fw	5′-ATGGACCAGATCCAGGTGAG-3′	Real time PCR for *lasA*
lasA.rv	5′-CGTTGTCGTAGTTGCTGGTG-3′	
lasB.fw	5′-ATCGGCAAGTACACCTACGG-3′	Real time PCR for *lasB*
lasB.rv	5′-ACCAGTCCCGGTACAGTTTG-3′	
lasI.fw	5′-TGCTCTGATCTTTTCGGA-3′	Amplication and labeling of lasI*
lasI.rv	5′-CGATCATCTTCACTTCCT-3′	probe used in EMSA
lasR-pGEX.fw	5′-CCGGATCCATGGCCTTGGTTGACGGTTT-3′	LasR amplification for cloning in pGEX6p expression vector
lasR-pGEX.rv	5′-CCGAATTCTCAGAGAGTAATAAGACCCA-3′	

### *In silico* Prediction of the Interaction of Naringenin With LasR

The LasR structures available on PDB were downloaded and confirmed incomplete. To avoid missing any potential interaction of the few missing amino acids, a full model of the structure of LasR was built using homology modeling with RaptorX (Kallberg et al., 2012) and used for subsequent docking. The structure of naringenin was optimized using Semiempirical Quantum Mechanics with OpenMopac (Stewart, 2008) using PM7 in an implicit water moiety and analyzed to assign partial charges prior to docking.

An initial docking step was carried out using PatchDock (Schneidman-Duhovny et al., 2005) with naringenin against the whole surface of the full-sequence LasR model to obtain 100 poses. After inspection, the best 20 were selected for further analysis and the complexes were subjected to Molecular Mechanics optimization using UCSF Chimera (Pettersen et al., 2004) with the AMBER ff14SB (Maier et al., 2015) force field allowing up to 1000 Newton-Raphson minimization steps and 100 conjugate-gradient steps to account for full flexibility in the interaction of LasR with naringenin.

Affinity was estimated using X-Score (Wang et al., 2002) and DSX (Neudert and Klebe, 2011), and UCSF Chimera was used to calculate the H-bonds, contacts, and clashes. Pose selection was based on docking scores, affinity estimates, counts of contacts and H-bonds, and absence of clashes. PDB structure 3ix3 was used to calculate the predicted affinity of LasR and 3OC12-HSL for comparison with naringenin.

The best interactions were selected to identify the preferential binding site, and this was used to perform a higher quality, restricted docking using AutoDock/Vina (Morris et al., 2009). The resulting poses were optimized and analyzed as described.

### Electrophoretic Mobility Shift Assays

The complete intergenic region upstream of *lasI* was amplified by using the fluorescein labeled oligonucleotides lasI.fw and lasI.rv (Table 2). The DNA (90 ng) was incubated with 20 μg of protein extracts of *E. coli* SHA011 cells or with 5 μg of purified LasR in the binding buffer (20 mM Tris-HCl pH 7.4, 1 mM EDTA, 150 mM KCl, 1 mM DTT, 10 μM 3OC12-HSL or DMSO, 100 μg/mL BSA, 50 ng/μL Poly (dIdC) and 10% glycerol). The inhibition assay was carried out by adding naringenin (10, 50, and 500 μM), or DMSO, to the binding reaction buffer during 30 min at room temperature. DNA-protein complexes were subjected to electrophoresis on 6% native polyacrylamide gels and visualized by using a Typhoon phosphorimager.

### *In vivo* Analysis of the Competition Between 3OC12-HSL and Naringenin Using a LasR-Based Reporter Strain

*In vivo* analysis of the potential competition between naringenin and 3OC12-HSL for their binding to LasR was performed using the *E. coli* JM109 (pSB1142) LasR-based reporter strain carrying the plasmid pSB1142 (Table 1). This plasmid contains the gene encoding LasR and the *luxCDABE* operon under control of the LasR-dependent *lasI* promoter. The reporter strain produces LasR, but light is emitted only when the protein is in its active form, in presence of the exogenously added effector 3OC12-HSL. Only when the effector is bound to LasR, the regulator is active, being able to recognize the *lasI* promoter, and activates the LasR-dependent expression of the *luxCDABE* operon. This system hence allows to get direct information on the *in vivo* activation of LasR through the binding of its effector and, consequently on the *in vivo* inhibition of such binding by potential 3OC12-HSL competitors as naringenin. To perform the assay, overnight biosensor cultures were diluted to 0.01 OD600 in LB into Flat white 96-well plates with optical bottom (Thermo Scientific Nunc) adding then the molecule(s) of interest. The assay was carried out by growing the LasR-based reporter strain in the presence of 1 μM of 3OC12-HSL, 1 μM of naringenin or both (competition assay). The capacity of each molecule to displace the other, already bound to LasR, was analyzed by adding one of the molecules at time zero and the other one with a delay of 2 h. Bioluminescence and OD600 were monitored at 37°C using a TECAN Infinite M200 plate reader. Luminescence was represented as relative light units normalized by growth (OD_600_).

### Gene Expression Analysis by Real Time PCR

*Pseudomonas aeruginosa* cells were grown in LB containing either 1.5 mM of naringenin or its solvent, DMSO. Independent cultures in which naringenin was added either at time 0 or 4 h after inoculation (early stationary phase) were grown. Cells were harvested, after 4 h of naringenin treatment, by centrifuging the cultures at 7000 rpm for 20 min at 4°C. The cell pellets were stored at −80°C and total RNA was extracted using the RNeasy minikit (Qiagen), according to the manufacturer’s instructions. Samples were treated with Turbo DNA-free (Ambion) to eliminate any remaining DNA. RNA integrity was verified on a 1% agarose gel, and the absence of DNA was verified by PCR using primers rplU.fw and rplU.rv (Table 2). cDNA was synthesized from RNA using a High Capacity cDNA reverse transcription kit (Applied Biosystems). Real time PCR was performed using a Power SYBR green kit (Applied Biosystems) as indicated by the manufacturer. The primers (Table 2) were used at a concentration of 400 nM. 50 ng of cDNA were used in each reaction. A denaturation step (95°C for 10 min), followed by 40 cycles of amplification (95°C for 15 s and 60°C for 1 min) was followed for amplification and quantification. Primers rplU.fw and rplU.rv were used to amplify the housekeeping gene *rplU*. All the primers were designed using Primer3 Input software; their specificity was tested by BLAST alignment against *P. aeruginosa* PAO1 genome from Pseudomonas Genome Database (Winsor et al., 2016) and their efficiency was analyzed by real time PCR using serial dilutions of cDNA. Differences in the relative amounts of mRNA for the different genes were determined according to the 2^–ΔΔCt^ method (Livak and Schmittgen, 2001). In all cases, the mean values for relative mRNA expression obtained in three independent triplicate experiments were considered.

### Elastase Activity and Pyocyanin Production

*Pseudomonas aeruginosa* cells were cultured in 20 ml of LB, at 37°C, containing either 4 mM of naringenin or its solvent, DMSO, as previously described (Vandeputte et al., 2011), with some modifications. Naringenin or DMSO was added to independent cultures either at time 0 or 4 h after inoculation (early stationary phase). Eight hours after inoculation (late stationary phase), 2 ml samples were collected from each culture and centrifuged for 10 min at 7000 rpm, and the supernatants were filtered using 0.2 μm filters (Whatman). Pyocyanin production was determined by measuring the OD_690_ of filtered supernatants. The elastase assay was adapted from Kessler and Safrin (2014); 1 ml of Congo Red elastin (Sigma-Aldrich) was added to 100 μl of each sample, and the mixture was incubated at 37°C and 250 rpm for 2 h. Subsequently, samples were centrifuged (10 min, 7000 rpm) and the OD_495_ of the filtered supernatants was determined. Three replicates of each condition were included in the analyses.

## Results

### The Transcriptional Regulatory Activity of LasR Depends on 3OC12-HSL Presence During Protein Expression

**FIGURE 1:**
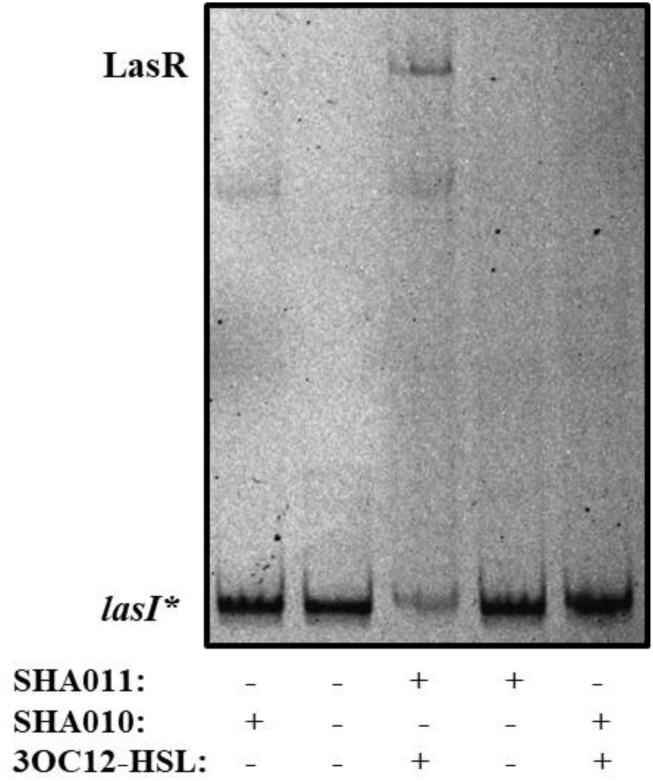
LasR DNA-binding capacity requires 3OC12-HSL during protein expression. *E. coli* SHA011 LasR producing strain was grown in the presence or absence of 20 μM 3OC12-HSL. As a control, *E. coli* SHA010, LasR non-producing strain was grown in same conditions. Protein extracts were tested for their capacity to bind to the lasI* probe by EMSA assays in the presence of 10 μM 3OC12-HSL. As shown, the presence of 3OC12-HSL in the culture is essential for purifying an active form of LasR (able to bind to the lasI* probe) from the *E. coli* SHA011 LasR producing strain. A non-specific retarded band was also observed in extracts obtained either from the control strain that does not produce LasR (SHA010) or from the SHA011 LasR-producing strain.

To analyze the putative capacity of naringenin to inhibit the binding of LasR to the *lasI* promoter, whose activity is directly dependent of LasR binding, we started by establishing the purification conditions required to obtain an active LasR protein. For that, the open reading frame of *lasR* was cloned into the inducible expression vector pGEX6p (Table 1), next to a glutathione *S*-transferase (GST) tag, therefore encoding a LasR-GST fusion protein. The resulting clones, containing either pGEX6p or pGEX6p-LasR (pSHA-LasR plasmid, see Table 1) were dubbed SHA010 and SHA011, respectively (Table 1). Although previous works had suggested that LasR purification requires the presence of its cognate signal effector 3OC12-HSL to obtain a fully active protein (Kiratisin et al., 2002; Schuster et al., 2004; Bottomley et al., 2007), biochemical studies would be far simpler using a purified protein without the bound activator. Therefore, we firstly expressed LasR from *E. coli* SHA011, either in presence or in absence of 20 μM of 3OC12-HSL. Protein extracts obtained in each of the conditions were used for performing electrophoretic mobility shift assays (EMSAs) using the complete intergenic region upstream of *lasI*, containing the LasR-binding site (Schuster et al., 2004), as the DNA probe (hereafter dubbed as *lasI*^∗^). As shown in Figure 1, only the protein extracts obtained in the presence of 3OC12-HSL presented the capacity to bind *lasI*^∗^. Moreover, addition of 3OC12-HSL to the binding reaction containing the inactive protein extracts, obtained from cultures grown without 3OC12-HSL, did not rescue the DNA-binding capability of LasR. This finding is in agreement with previous information regarding the conditions required to purify LasR and reasserts the requirement of 3OC12-HSL during LasR production to stabilize the conformation of the nascent protein and to permit its dimerization (Schuster et al., 2004; Bottomley et al., 2007), hence preventing the formation of insoluble aggregates and the subsequent degradation by endogenous proteases (Zhu and Winans, 2001; Kiratisin et al., 2002). Further, our results support that the 3OC12-HSL effector is unable to turn on the activity of an inactive LasR protein. This information is in agreement with those reported in a recent publication, which states that, as other LuxR-type regulators (Zhu and Winans, 2001; Oinuma and Greenberg, 2011), LasR is only soluble when ligand is present (Mccready et al., 2019). Further, it has been also shown that extensive dialysis of active LasR against signal-free buffer does not allow to remove such signal from the protein, hence impeding to have purified LasR without any bound effector site (Schuster et al., 2004). Taking into account this situation, LasR purified in the presence of its effector, which forms the LasR-3OC12-HSL complex, was used in further biochemical assays.

### Naringenin Inhibits the Binding of LasR to the *lasI* Promoter

The structural basis of the molecular interactions of 3OC12-HSL with LasR have been studied by analyzing the co-crystal structure of a C-terminally truncated form of LasR bound to this effector (Bottomley et al., 2007). Previous efforts to get soluble forms of LasR were not successful in line with the reported requirement of 3OC12-HSL during LasR production for stabilizing the protein (Schuster et al., 2004). Further work allowed a better delimitation of the structure of the effector binding site at 1.4 Å of resolution (Zou and Nair, 2009). To address whether or not naringenin could be a competitor of 3OC12-HSL, we analyzed *in silico* the capability of LasR to accommodate the flavonoid in its binding pocket.

**FIGURE 2:**
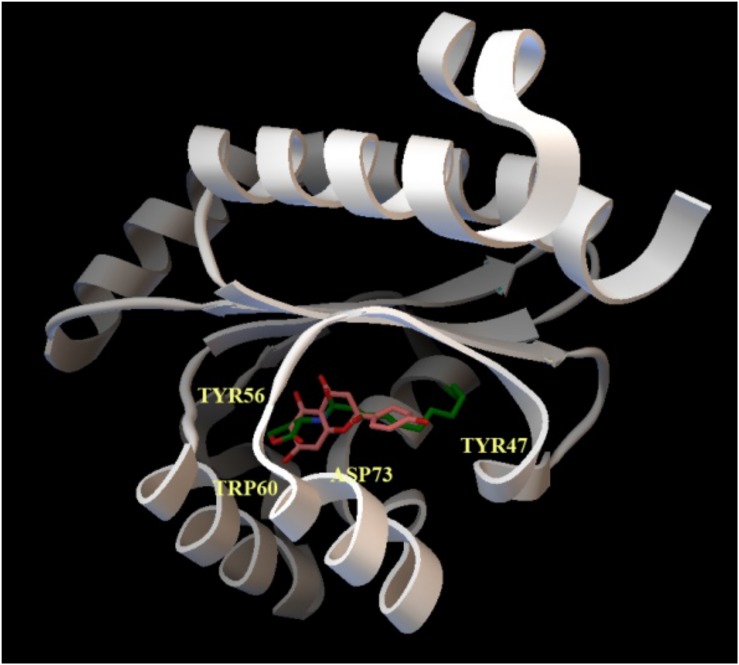
Docking of naringenin and 3OC12-HSL to LasR. The cartoon representation shows the best binding conformation for the analyzed ligands, naringenin (orange) and 3OC12-HSL (green), in the ligand binding pocket of the protein. In yellow, the aminoacid residues Tyr56, Trp60 and Asp73, which interact with the polar homoserine lactone head group of the autoinducer and with the hydrocarbon chain of the autoinducer (Tyr47), are shown. The prediction model supports that naringenin may be accommodated in the binding pocket where 3OC12-HSL binds, suggesting a possible competition of both molecules for LasR binding.

To explore this issue, we obtained a full-sequence homology model of LasR using Raptor-X (see section “Materials and Methods”) to avoid ignoring any potential interaction between LasR and naringenin, and docked naringenin to the model. Docking with PatchDock (see section “Materials and Methods”) revealed a number of potential binding sites for naringenin all over the surface of LasR. To evaluate their relative preference, we optimized the best poses using Molecular Mechanics to allow full flexibility on both molecules (see section “Materials and Methods”). Affinity estimations revealed similar rankings with both DSX and X-Score, with the first five poses consistently showing a significantly larger score than the rest and binding the active pocket of LasR. Docking with AutoDock/Vina specifically to the identified preferred binding site, resulted in higher quality conformers (Figure 2) according to the subsequent analysis. The affinity estimated for the best naringenin binding pose to LasR is similar to the affinity estimated from the structure of 3OC12-HSL bound to LasR, determined experimentally by X-ray crystallography (−9.30 kcal/mol for naringenin and −9.25 kcal/mol for 3OC12-HSL, according to X-Score). Despite seemingly showing some freedom to position itself in the binding pocket, our analysis revealed that, in any case, naringenin would be unable to interact with all the amino acids required for the formation of an oligomerizable LasR monomer.

**FIGURE 3:**
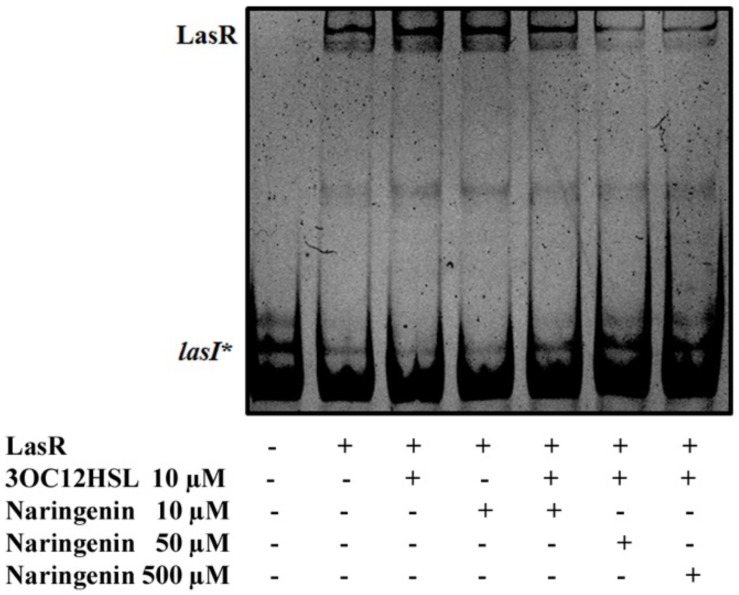
Partial inhibition of LasR binding activity by naringenin. The EMSA assays were carried out using a purified active protein obtained from SHA011 strain grown in the presence of 3OC12-HSL. The capacity of naringenin to displace 3OC12-HSL from LasR was analyzed by adding increasing concentrations of the flavonoid (10, 50, 500 μM) to the binding reaction. The figure shows that neither 3OC12-HSL (10 μM) nor naringenin (10 μM) addition to the binding buffer alters the LasR-DNA binding complex. However, increased concentrations of naringenin (50 and 500 μM) in the binding buffer were able to partially inhibit the LasR-DNA interaction, even in the presence of 3OC12-HSL (10 μM), evidencing that the flavonoid may partially displace the 3OC12-HSL from LasR.

To verify this prediction, we purified LasR from *E. coli* SHA011 grown in the presence of 20 μM 3OC12-HSL, condition where LasR presented an active conformation when protein extracts were analyzed (Figure 1). The protein was purified using a GST-affinity column and the LasR protein was collected by removing the GST-tag as described in Section “Materials and Methods.” The purified non-tagged protein appears as a single band when analyzed by polyacrylamide gel electrophoresis (PAGE) and was identified as LasR by MALDI-TOF mass spectrometry. Afterward, we measured the capacity of purified LasR for binding to *lasI*^∗^ by EMSA and determined the capacity of increasing concentrations of naringenin to inhibit this binding. As shown in Figure 3, LasR purified from *E. coli* SHA011 grown in presence of 3OC12-HSL is able to bind its cognate DNA operator. Moreover, the addition of 10 μM 3OC12-HSL to the binding reaction buffer does not increase LasR-DNA binding capacity, supporting that the protein is fully active (Figure 3). To analyze the capacity of naringenin to displace 3OC12-HSL from LasR, we added increasing naringenin concentrations, from 10 through 50 and reaching 500 μM, to the EMSA binding reaction. As Figure 3 shows, the addition of 50 μM naringenin to the binding buffer reduces the amount of the DNA-LasR complex indicating that the flavonoid partially displaces 3OC12-HSL from LasR. Nevertheless, concentrations of naringenin as high as 500 μM do not further reduce the amount of LasR-DNA complex. This finding indicates that the ability of naringenin to outcompete 3OC12-HSL is limited when the latter is previously bound to the LasR protein, a feature in agreement with the *in silico* analysis, which predicts that the affinities of LasR for naringenin and O3C12-HSL are similar.

### Perception of 3OC12-HSL by LasR Is Inhibited by Naringenin

Our *in vitro* results indicate that, once 3OC12-HSL is bound to LasR, naringenin competes just partially for such binding (Figure 3), being unable to completely displace the AHL. To analyze the capability of both molecules to displace each other when the first molecule is bound to LasR, the LasR-based reporter strain, *E. coli* JM109 (pSB1142) (Table 1), was used. This is a 3OC12-HSL non-producer strain that harbors the pSB1142 plasmid containing the *lasR* gene, which encodes the QS response regulator LasR, and a *luxCDABE* reporter operon under the control of the LasR-dependent *lasI* promoter. This biosensor strain produces LasR, but the protein is active just in presence of 3OC12-HSL. Only when this effector in bound to LasR, the protein binds to the *lasI* promoter and triggers the transcription of the *lux* system. Therefore, luminescence emitted from LasR-based biosensor strain provides a direct *in vivo* quantification of LasR activation by its effector and, conversely, when a potential inhibitor as naringenin is added, provides a direct measurement of the competition for LasR of the activator and the inhibitor. To note here that, since LasR cannot be purified in its inactive form, in the absence of an effector, biosensors as the one herein used are regularly utilized for studying the interaction of LasR with potential effectors (Mccready et al., 2019).

**FIGURE 4:**
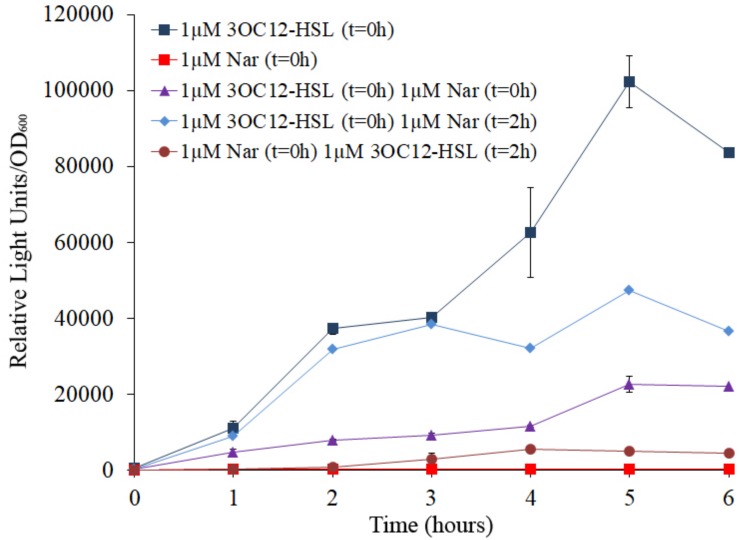
Analysis of naringenin and 3OC12-HSL competition for the binding of LasR using a biosensor strain of *E. coli*. The *E. coli* JM109 (pSB1142) LasR-based reporter strain was used to analyze the capacity of naringenin and 3OC12-HSL to displace each other when the first molecule has already bound the LasR regulator. The luminescence produced by *E. coli* JM109 (pSB1142) was recorded and it is represented as relative light units normalized by growth (OD_600_). 3OC12-HSL or naringenin were added in combination or separately, at different times. No differences in growth were observed between the analyzed conditions. As shown, the highest or the lowest luminescence were emitted in presence of 1 μM of 3OC12-HSL or 1 μM naringenin, respectively, when were independently added at time zero (*t* = 0 h). Moreover, intermediate luminescence was observed when the two compounds were added at time zero, demonstrating that there is a competition between them for LasR binding. Addition of 3OC12-HSL after 2 h of incubation with naringenin (added at time = 0 h) was unable to increase LasR activity, while addition of naringenin after 2 h of incubation with 3OC12-HSL (added at time = 0 h) was enough to partially decrease the LasR-dependent luminescence emitted by *E. coli* JM109 (pSB1142). Altogether, these results suggest a more efficient displacement of 3OC12-HSL by naringenin compared with the displacement of naringenin by 3OC12-HSL. Error bars represent standard error of three independent replicates.

As could be expected, when *E. coli* JM109 (pSB1142) cells were grown in the presence of 3OC12-HSL, induction of the *luxCDABE* reporter was observed, while no induction was observed when *E. coli* JM109 (pSB1142) cells were grown in the presence of naringenin (Figure 4). It is important to remark that the concentrations of naringenin used in the assay do not alter the growth of the reporter *E. coli* strain. When, *E. coli* JM109 (pSB1142) cells grown in presence of equimolar concentrations of naringenin and 3OC12-HSL, showed a relevant reduction of the expression of the reporter operon, compared to the luminescence levels reached when cells were grown with 3OC12-HSL alone. This result confirms that naringenin can inhibit the activity of LasR *via* its competition with 3OC12-HSL. However, our *in vitro* results (Figure 3) suggested that naringenin just partially outcompetes 3OC12-HSL when this AHL is already bound to LasR protein. To analyze this issue, the *E. coli* JM109 (pSB1142) strain was grown in presence of 3OC12-HSL and, with a delay of 2 h, naringenin was added to the cultures. As shown in Figure 4, the inhibitory effect of naringenin, when added to cultures already containing 3OC12-HSL, was lower than when both compounds were simultaneously added, while no induction of the LasR-driven response was observed when 3OC12-HSL was added with 2 h of delay respect to naringenin addition. In agreement with our *in vitro* analyses (Figure 3), these results suggest that, although a partial shift between 3OC12-HSL and naringenin is possible when the first is bound to LasR, the efficiency of this displacement is low. Altogether, our results support that QS inhibition mediated by naringenin is more efficient when naringenin binds to the nascent LasR protein than when 3OC12-HSL has already bound to the LasR regulator. This is consistent with the similar affinities estimated for both molecules during modeling.

### The Expression of QS-Regulated Genes and the Production of QS-Controlled Virulence Factors in *P. aeruginosa* Are Inhibited in a Time-Dependent Manner by Naringenin

The previous analyses show that the pre-activation of LasR by 3OC12-HSL may limit the inhibitory capacity of naringenin. These analyses were made *in vitro* and in a heterologous host that does not produce QS signals, in order to fully control the concentrations and timing of both, naringenin and 3OC12-HSL addition.

**FIGURE 5:**
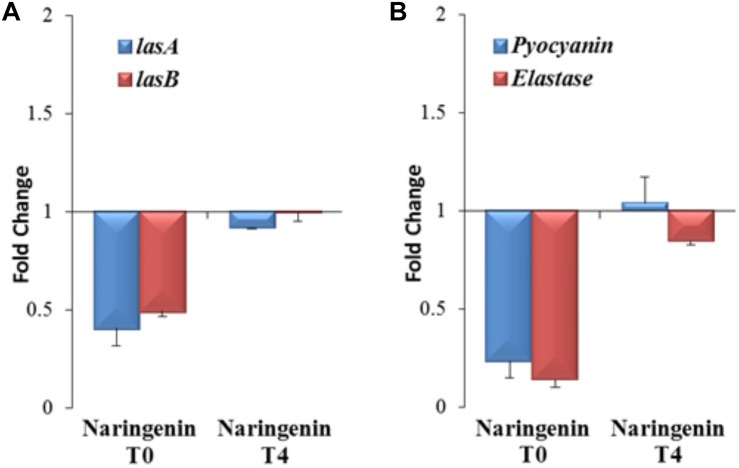
Analysis of early and late inhibition of the expression of the QS-regulated genes and virulence factors by naringenin in *P. aeruginosa* PAO1. **(A)** The expression of the *lasA* and *lasB* QS-regulated genes was analyzed by real time PCR in cells treated with naringenin at *t* = 0 h (early inhibition) or at *t* = 4 h (late inhibition) after 4 h of growth. Results are represented as the fold change in the level of expression compared to same strain grown in presence of the solvent of naringenin, DMSO. The results show that inhibition of LasR-dependent regulation by naringenin is more efficient when this flavonoid is added at *t* = 0 h. Contrary, there is no inhibition by naringenin of *lasA* and *lasB* expression when this flavonoid was added 4 h after incubation (early stationary phase), when 3OC12-HSL production by *P. aeruginosa* is high. **(B)** Elastase activity and pyocyanin production were determined at the late stationary phase (OD_600_ = 4.4; *t* = 8 h) in cells treated, at *t* = 0 h (early inhibition) or at *t* = 4 h (late inhibition), with naringenin or its solvent, DMSO. These graphs show that inhibition of LasR-dependent regulation and QS-dependent production of virulence factors by naringenin is more efficient when this flavonoid is added at *t* = 0 h, a cellular status where the production of 3OC12-HSL is practically zero. Contrary, there is no inhibition by naringenin of QS-regulated genes or of virulence factors production when this flavonoid was added 4 h after incubation (early stationary phase), when 3OC12-HSL production by *P. aeruginosa* is high and naringenin cannot displace the AI from LasR. In both figures, error bars represent standard error of three independent replicates.

Nevertheless, since 3OC12-HSL is naturally produced by *P. aeruginosa* depending on the cellular status of the population, the functional verification of these results in this organism, the host bacterium of the studied QS network, is needed. Consequently, the expression of the QS-regulated genes *lasA* and *lasB*, that encode a staphylolysin and a pseudolysin respectively (Storey et al., 1998), was measured using early or late QS inhibitory conditions in *P. aeruginosa* to determine if the inhibitory capacity of naringenin is also time-dependent in this microorganism. For that purpose, *P. aeruginosa* PAO1 was grown in the presence or in the absence of naringenin, added at time zero (early inhibition), when 3OC12-HSL concentration is low, or after 4 h of growth (late inhibition), when the population is at early stationary phase and cells have accumulated 3OC12-HSL. In both inhibitory assays, gene expression was measured by real time RT-PCR after 4 h of naringenin treatment. As shown in Figure 5A, naringenin was able to reduce halfway the expression of both *lasA* and *lasB* genes in early inhibitory conditions. However, late inhibition of the expression of these QS-regulated genes was not observed.

It has been described that naringenin is able to inhibit the production of QS-regulated virulence factors in *P. aeruginosa* (Rasamiravaka et al., 2013). However, this work neither analyze the molecular basis of such inhibition nor its time-dependent efficacy. According to our results, this should be also relevant for efficient QS inhibition, because the inhibition of the QS response may depend on the QS status of the population. Consequently, we analyzed the effect that either early (*t* = 0 h) or late (*t* = 4 h) addition of naringenin may have on the production of two known QS-regulated virulence factors, pyocyanin and elastase, at the late stationary phase. As shown in Figure 5B, early addition of naringenin inhibited pyocyanin and elastase production, up to 5- and 7-times, respectively. However, when naringenin was added later, inhibition of the production of these virulence factors was not detected (Figure 5B).

Altogether, these results indicate that early inhibition of the QS response mediated by naringenin is possible in *P. aeruginosa*, but its effectiveness may be compromised when 3OC12-HSL is already bound to the LasR regulator.

## Discussion

Flavonoids play important physiological functions in plants as well as in plant-bacteria interactions, in particular in the natural ecosystem of the rhizosphere, where microorganisms live in contact with plants’ roots. In this environment, plant-produced flavonoids confer to them protection against microbial attacks (Farhadi et al., 2019). One of such protection mechanism has been described in the case of *Pseudomonas syringae* infections; flavonoids reduce bacterial virulence by inhibiting the GacS/GacA two-component system (Chatterjee et al., 2003; Vargas et al., 2013). Meanwhile, in an example of Red-Queen adaptive co-evolution, this bacterium is able to overcome this inhibition by the flavonoid-dependent induction of the multidrug efflux system, MexAB-OprM (Vargas et al., 2011; Alcalde-Rico et al., 2016), which is able to extrude flavonoids and is needed for the efficient colonization of tomato plants by this bacterium. Thus, taking into consideration the intense co-evolution of plants and bacteria, exemplified by the above described flavonoid-mediated plant/bacteria interactions, it is not difficult to think on the existence of plant molecules with capacity to inhibit one of the most relevant circuits regulating bacterial virulence, the QS regulatory network, a field currently under study (Ta et al., 2014; Bacha et al., 2016; Dettweiler et al., 2019).

It has been described that naringenin is a flavonoid with therapeutic potential (Salehi et al., 2019), particularly in inflammation-related diseases (Zeng et al., 2018), as well as an inhibitor of the production of *P. aeruginosa* QS-regulated virulence factors (Vandeputte et al., 2011; Rasamiravaka et al., 2013). However, the molecular mechanism by which naringenin inhibits QS in *P. aeruginosa* has not been determined yet. The modeling of naringenin binding to LasR predicts that this flavonoid binds preferentially to the same site as 3OC12-HSL, with an affinity that seems similar to that of 3OC12-HSL, and that it would be unable to establish contact with all the amino acid residues needed for the formation of the structure of a functional monomer that can oligomerize (Figure 2), so the nascent LasR chain that binds naringenin would be unable to oligomerize, and 3OC12-HSL would not be able to form active LasR complexes in the presence of this flavonoid (Figure 4). Further, the similarity in affinities could explain why each of the two molecules has difficulty in displacing the other once bound to LasR, since it has been stated that only ligands presenting higher affinities can displace from LasR the originally bound ligand (Paczkowski et al., 2017). Although a direct effect of naringenin on LasR stability cannot be fully disregarded, our experimental findings support that naringenin inhibits *P. aeruginosa* QS response through its binding to the LasR regulator by a time-dependent model of competition with 3OC12-HSL. This model implies that, when *P. aeruginosa* populations present low densities (and hence low 3OC12-HSL concentration), naringenin can bind to nascent LasR, inhibiting its 3OC12-HSL-dependent activation. However, in dense *P. aeruginosa* population, presenting a high accumulation of 3OC12-HSL, inhibition of the QS response by naringenin would not be achieved. This time-dependent model of inhibition might compromise the efficiency of this flavonoid as a QS inhibitor, hence making difficult its application in clinics, but also opens the door for seeking more efficient analogs. This issue will be relevant when dense populations, whose QS response is already triggered, as those forming biofilms (Costerton, 2001; Winstanley et al., 2016), are confronted with this type of QS inhibitors.

Deepening into the mechanisms of QS inhibition may help in the development of such inhibitors, which have been proposed as suitable anti-infective drugs (Fleitas Martinez et al., 2018). Based in our results, we therefore propose that screenings for QS inhibitors should not only focus on finding molecules with an overall inhibitory effect of virulence phenotypes, but also should analyze their effectiveness over dense populations and select those whose mechanisms of action do not depend on the QS status of populations. This would increase their potential application against *P. aeruginosa* infections, where QS response is frequently triggered.

## Data Availability Statement

All datasets generated for this study are included in the article/supplementary material.

## Author Contributions

SH-A, MA-R, and TG-G performed the experimental work. JV performed the computational predictive work. SH-A, MA-R, TG-G, JV, and JM designed the study, contributed to the interpretation of the results and in writing the article.

## Conflict of Interest

The authors declare that the research was conducted in the absence of any commercial or financial relationships that could be construed as a potential conflict of interest.
